# Cold acclimation threshold induction temperatures of switchgrass ecotypes grown under a long and short photoperiod

**DOI:** 10.1111/ppl.13812

**Published:** 2022-11-22

**Authors:** Ian R. Willick, David B. Lowry

**Affiliations:** ^1^ Department of Plant Biology Michigan State University East Lansing Michigan USA; ^2^ Great Lakes Bioenergy Research Center Michigan State University East Lansing Michigan USA; ^3^ Plant Resilience Institute Michigan State University East Lansing Michigan USA; ^4^ Kentville Research and Development Centre Agriculture and Agri‐Food Canada Kentville NS Canada; ^5^ Department of Ecology, Evolutionary Biology, and Behavior Michigan State University East Lansing Michigan USA

## Abstract

Plants can cold acclimate to enhance their freezing tolerance by sensing declining temperature and photoperiod cues. However, the factors influencing genotypic variation in the induction of cold acclimation are poorly understood among perennial grasses. We hypothesized that the more northern upland switchgrass (*Panicum virgatum* L.) ecotype develops a higher degree of freezing tolerance by initiating cold acclimation at higher temperatures as compared with the coastal and southern lowland ecotypes. First, we determined the optimal method for assessing freezing tolerance and the length of exposure to 8/4°C required to induce the maximum level of freezing tolerance in the most northern upland and most southern lowland genotypes. We characterized the maximum freezing tolerance of eight uplands, three coastal and five lowland genotypes grown for 21 days at 8/4°C and a 10 or 16 h photoperiod. Next, we identified the temperature required to induce cold acclimation by exposing the 16 genotypes for 7 days at 20–6°C constant temperatures under a 10 or 16 h photoperiod. Cold acclimation initiated at temperatures 5 and 7°C higher in upland than in coastal and lowland genotypes. Among upland genotypes the shorter photoperiod induced cold acclimation at a 1°C higher temperature. Genotypes originating from a more northern latitude initiate cold acclimation at higher temperatures and develop higher maximum freezing tolerances. An earlier response to declining temperatures may provide the upland ecotype with additional time to prepare for winter and provide an advantage when plants are subjected to the rapid changes in fall temperature associated with injurious frosts.

## 1 INTRODUCTION

The response of a plant to a combination of declining temperature and photoperiod is intrinsically related to the cessation of growth and the capacity of perennating organs to cold acclimate. During higher fall and winter temperatures, perennial species will extend the growing season and delay fall senescence (Ergon, 2017; Jeong et al., 2011). This uncoupling of short‐day length from cooler nighttime temperatures could disrupt the senescence of aboveground foliage and the induction of cold acclimation. Experiments are required to establish the relative importance of temperature and photoperiod for acclimation to facilitate a more targeted mechanistic understanding of the phenological response of a plant to increasing temperature (Gusta & Wisniewski, 2013; Hänninen et al., 2019).

The induction of cold acclimation in perennial plants is divided into three key stages (Weiser, 1970). In the first stage of cold acclimation, reduced growth rates coincide with declining fall day length, night‐time temperatures, and a reduction in the ratio of red to far‐red light. Exposure to threshold low induction temperatures induces the second stage of cold acclimation that results in a cessation of vegetative growth and drives an increase in the acquisition of freezing tolerance (Fowler, 2008). Some perennials and winter annuals exposed to nonlethal freezing temperatures undergo the third stage of cold acclimation, historically known as frost hardening (Weiser, 1970) and contemporarily referred to as sub‐zero acclimation (Takahashi et al., 2021). In grasses, sub‐zero acclimation can enhance the freezing survival of perennating organs for a period of weeks to months (Willick et al., 2021). Exposure of grasses to higher fall temperatures increases the vulnerability of a plant to fall and winter injury by delaying the induction of secondary cold acclimation and reducing the maximum attainable level of freezing tolerance obtained following the third stage of cold acclimation.

Photoperiod and temperature are important cues for cold acclimation in perennial plants (Ergon, 2017; Kalcsits et al., 2009; Li et al., 2003; Malyshev et al., 2014). Some woody perennials require only declining photoperiod to induce a cold acclimation response while others are primarily driven by temperature (Chang et al., 2021; Cooke et al., 2012; Tanino et al., 2010). The relative influence of temperature and photoperiod could determine the extent to which climate warming in the fall will alter the timing and rate of cold acclimation. Since photoperiod cycles will remain constant as a given location, it is important to know how plants will respond to the uncoupling of traditional low temperature and short photoperiod cues. For hybrid aspen (*Populus tremula* × *Polulus tremuloides* Michx.) (Welling et al., 2002) and silver birch (*Betula pendula* Roth) (Li et al., 2003) a short photoperiod and low threshold temperature interact synergistically to enhance freezing tolerance.

Overall trends have emerged in the woody perennial literature regarding the importance of a short‐day photoperiod to initiate plant senescence (Lagercrantz, 2009). However, the relative importance of photoperiod in cold acclimation varies in northern as opposed to southern ecotypes (Howe et al., 1995; Kalcsits et al., 2009; Sarath et al., 2014; Smithberg & Weiser, 1968; Tanino et al., 2010). Compared to woody perennials (Howe et al., 1995; Kalcsits et al., 2009; Li et al., 2003; Smithberg & Weiser, 1968; Tanino et al., 2010), the factors influencing genotypic variation in the induction of cold acclimation are less understood for perennial grasses (Malyshev et al., 2014; Sarath et al., 2014; Wingler, 2015). In perennial temperate grasses, the insufficient translocation of aboveground carbon (Zegada‐Lizarazu et al., 2012) and nitrogen (Yang et al., 2016) to the overwintering crowns, rhizomes, and associated tiller buds can significantly reduce winter survival. Perennial grasses adapted to northern temperate climates around the time of flowering translocate aboveground nutrients to belowground perennating organs (Ergon, 2017; Sarath et al., 2014; Vogel et al., 2011). The more southern‐adapted perennial temperate grasses develop aboveground biomass until exposure to sub‐lethal freezing temperatures (Sarath et al., 2014). While there is significant genetic variation for overwintering survival among perennial grasses (Casler et al., 2004, 2007; Malyshev et al., 2014), it is not clear what role the variation in cold acclimation plays in driving that pattern. Therefore, it is important to understand how cold acclimation varies within a grass species to promote overwintering survival.

In the central United States, the rates of fall temperature and photoperiod declines covary with latitude (Hut et al., 2013; Lowry et al., 2019). As such, the variation in the induction of cold acclimation cues should be distributed among plant populations along a north–south cline. Switchgrass is an ideal system to study geographic differences in cold acclimation. It is a C4 perennial bunchgrass used as a bioenergy feedstock (Gelfand et al., 2013) with a large geographic range stretching from central Mexico to southern Canada. Most of the research in this system has focused on large differences in morphology and physiology between lowland and upland ecotypes (Casler et al., 2004, 2007; Lowry et al., 2014; Porter Jr, 1966). In contrast to the lowland ecotype, that is, generally restricted to Mexico and the southern half of the United States, the upland ecotype's distribution extends into Canada and has a comparatively higher tolerance to freezing injury (Casler et al., 2004; Poudel et al., 2019, 2020). In addition, a less‐studied coastal ecotype occurs primarily along the eastern seaboard of the United States. The coastal ecotype is similar to the lowland ecotype in terms of overwintering survival and plant architecture but displays upland leaf characteristics (Lovell et al., 2021).

In common garden experiments, greater overwintering survival was observed among the more northern upland switchgrass populations and declined along a latitudinal gradient (Casler et al., 2004). Lovell et al. (2021) observed 42% winterkill in lowland and coastal populations but only 2% winterkill in upland populations planted at sites in the northeastern United States. Quantitative trait loci analysis of a heterogeneous F_2_ population derived from the upland Summer and lowland Ellsworth identified cold response genes (*COR47*) and heat shock proteins (*HSP70*) affiliated with enhanced freezing tolerance (Poudel et al., 2019). A recombinant four‐way mapping population derived from two upland (DAC6 and VS16) and two lowland genotypes (AP13 and WBC3) (Milano et al., 2016) developed 2.1% mortality rate across 10 field sites as compared with a 14.5% mortality rate among the grandparental genotypes (Lowry et al., 2019). Southern genotypes AP13 and WBC3 experienced an 80% mortality rate at the most northern field site in Brookings, South Dakota due to winterkill (Lowry et al., 2019). While the majority of research in switchgrass has focused on overwintering survival as a general trait (Casler et al., 2004, 2007; Hope & McElroy, 1990; Lovell et al., 2021; Lowry et al., 2019), less is known about how upland, coastal, and lowland switchgrass enhance freezing tolerance in response to declining photoperiod and temperature.

**FIGURE 1:**
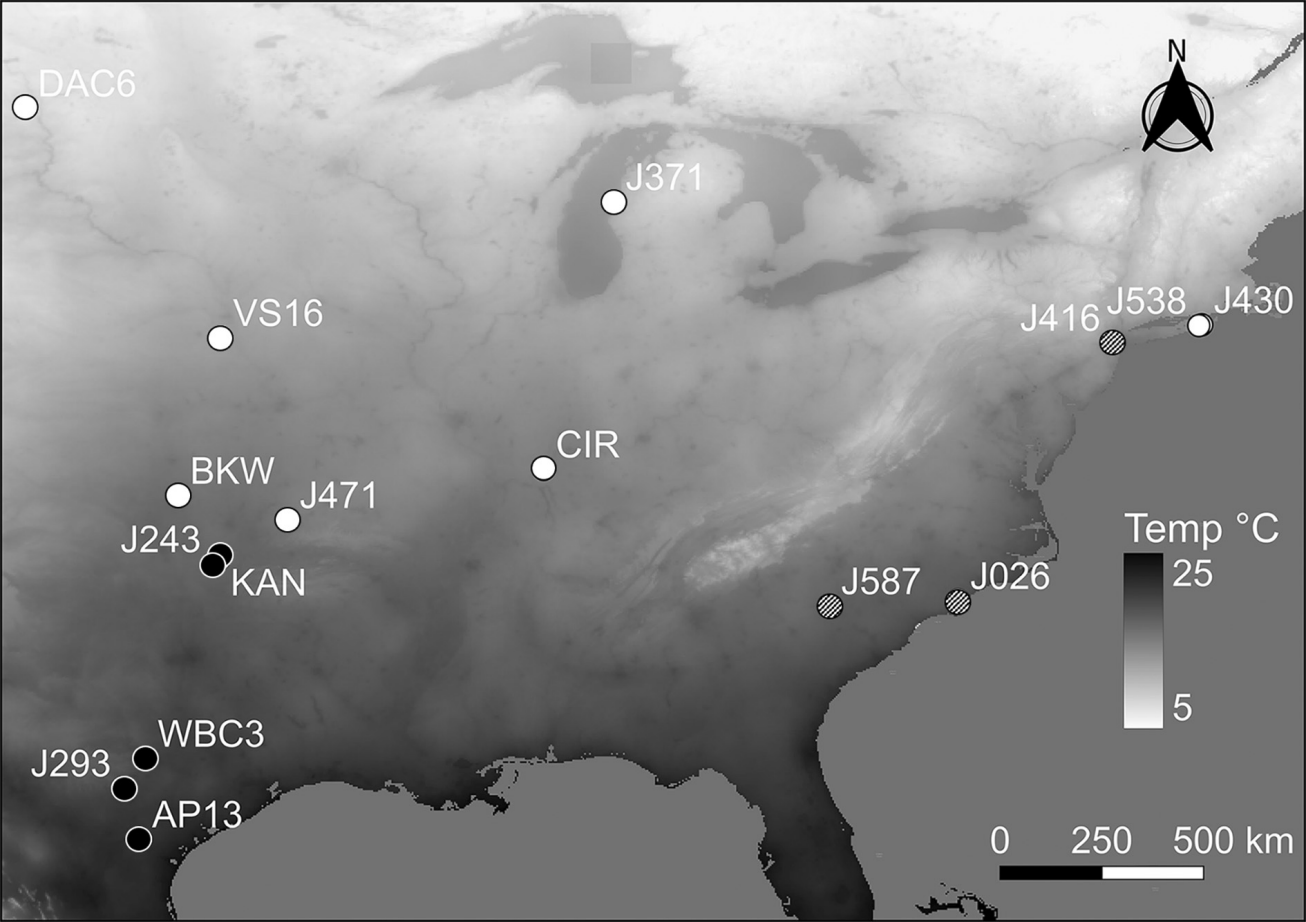
Map displaying the origins of the eight upland (open circle), three coastal (hashed circle), and five lowland (closed circle) switchgrass genotypes. Monochromatic shading illustrates differences in the minimum mean surface temperature (°C) for the month of September, as extracted from the WorldClim 2.0 historical data set (Fick & Hijmans, 2017). Gray coloration over oceans do not represent temperature differences and only provide visual contrast with land masses.

The objectives of our study were to conduct a freezing‐tolerance screening of the most northern upland genotype (DAC6) and most southern lowland genotype (AP13) to identify the optimal duration of low‐temperature exposure that induces maximum freezing tolerance. Using these optimized protocols, eight uplands, three coastal and five lowland genotypes will be characterized for maximum freezing tolerance and the temperature required to induce a cold acclimation response when exposed to either a long‐ (16 h) or short‐day (10 h) photoperiod (Figure 1). We hypothesize that the upland genotypes develop a higher degree of freezing tolerance by beginning to cold acclimate at higher temperatures as compared with the coastal and lowland genotypes.

## 2 MATERIALS AND METHODS

### 2.1 Plant cultivation

Sixteen switchgrass genotypes derived from N 46.39° to 28.33° latitude were included in this study (Table 1). Across this geographic range, temperature decreased while mean winter precipitation increased with latitude. The more northern genotypes originated from relatively cool‐moist conditions, whereas southern genotypes originated from warm‐arid conditions. All genotypes were clonally propagated at the Michigan State University Greenhouses (43.72°N, −84.47°W) by repeated division of individual parents. Genotypes AP13, DAC6, VS16, CIR, KAN, and BKW were, respectively, derived from the cultivars Alamo, Dacotah, Summer, Cave‐in‐Rock, Kanlow, and Blackwell are genetically similar to their prairie remnant populations (Casler et al., 2007). Ten genotypes (WBC3, J371, J430, J538, J416, J471, J243, J026, J587, and J293) were clones of individual switchgrass collected from native populations.

**TABLE 1:** Geographic origin, ecotype (E; upland [U], coastal [C] and lowland [L]) and climate data for the 16 switchgrass genotypes (Geno.) included in this study.

Geno. [E]	LAT (°)	LON (°)	Max_Sep_ (°C)	Min_Sep_ (°C)	Max_Oct_ (°C)	Min_Oct_ (°C)	Max_Nov_ (°C)	Min_Nov_ (°C)
DAC6 [U]	46.39	−100.94	23.5	7.5	15.7	0.7	4.1	−7.2
J371 [U]	44.08	−86.44	21.2	10.8	14.6	5.2	7.5	−0.1
J430 [U]	41.04	−71.93	22.4	13.8	17.1	8.2	12.1	3.8
J538 [U]	41.02	−72.00	22.0	13.5	16.7	8.0	11.7	3.5
VS16 [U]	40.72	−96.12	25.8	11.7	19.3	4.8	9.5	−2.2
J416 [C]	40.60	−74.13	24.5	14.0	18.4	7.7	12.5	3.1
CIR [U]	37.47	−88.16	27.7	14.7	21.6	8.0	14.4	3.3
BKW [U]	36.82	−97.18	29.0	15.9	22.7	9.0	14.3	2.3
J471 [U]	36.22	−94.48	27.6	14.8	21.9	8.3	14.5	2.5
KAN [L]	35.26	−96.18	29.7	16.4	23.9	10.0	16.5	4.0
J243 [L]	35.13	−96.32	29.7	16.5	24.0	10.1	16.5	4.0
J026 [C]	34.22	−77.94	29.2	18.6	24.7	12.1	20.3	7.3
J587 [C]	34.09	−81.12	29.5	17.2	24.3	10.4	19.3	5.5
WBC3 [L]	30.32	−97.98	31.2	19.0	26.6	13.8	20.4	8.2
J293 [L]	29.59	−98.54	31.6	19.8	27.2	14.6	21.4	9.0
AP13 [L]	28.33	−98.12	33.4	20.8	29.5	16.0	24.3	11.0

*Note*: Historical climate data (1970–2000) from WorldClim 2.0 was accessed using qGIS (vers. 2.24) to approximate mean maximum (Max) and minimum (Min) surface temperatures for the months of September (S), October (O) and November (N). This period is when switchgrass would begin to cold acclimate. Genotypes BKW and CIR are octoploid, whereas all other genotypes are tetraploid.

Propagules were transplanted in 10 cm square pots containing a 1:1 mixture of Sure Mix potting soil (Michigan Grower Products Inc., Galesburg, MI, USA) and Turface MVP calcined clay (Turface Athletics, Buffalo Grove, IL, USA) with a time‐release fertilizer (Osmocote 14‐14‐14, The Scotts Company, Marysville, OH, USA). Switchgrass pots were transferred to a drip‐irrigated greenhouse held at 28°C with 70% relative humidity and a light intensity at the canopy level of 700 μmol photons m^−2^ s^−1^ supplemented with high‐pressure sodium lighting. Prior to experiments, switchgrass pots with a minimum of four developing tillers were acclimatized to the environmental conditions in growth cabinets (BioChambers, Winnipeg, MB, Canada) for 21 days at 25/20°C set at 70% relative humidity with a 16 h photoperiod and a canopy light intensity of 700 μmol photons m^−2^ s^−1^.

### 2.2 Injury in rhizomes frozen prior to or following excision

Northern upland DAC6 and southern lowland AP13 were held at 8/4°C for 21 days prior to assessment of injury using one of the three following tests: (1) Whole‐plant recovery. (2) Injury in rhizomes frozen prior to excision from whole plants. (3) Injury in rhizomes frozen following excision from whole plants.

For assessment of injury in whole plants and intact rhizomes, 10 switchgrass pots per test temperature were transferred to a programmable freezer (Thermotron 8200, Thermotron Industries, Holland, MI, USA) set at 0°C for 1 h. The temperature within the freezer was cooled to −2°C over 1 h, held at −2°C for 12 h and then cooled to −4°C over 1 h. Aboveground tissues were misted with a water solution containing clay particulate and ice nucleation active bacteria collected in October 2020 from native grasses near the Michigan State University Greenhouses to promote uniform ice nucleation among samples. The ice nucleation solution maintained an average ice nucleation temperature of −3.7 ± 0.4°C (*N* = 50). The programmable freezer was then cooled 2°C h^−1^ to three predetermined test temperatures that were 2°C apart. Pots were transferred to a dark room set at 5°C for 24 h and then to a greenhouse room maintained at conditions for plant cultivation.

For whole‐plant recovery, aboveground biomass was trimmed after 7 days in the greenhouse to within 24 cm from the soil. Survival was scored after 28 days as the proportion of switchgrass pots that regrew tillers. The LT_50_ was calculated from survival curves (Willick et al., 2021) and the experiment was repeated four times for each genotype and treatment combination. To assess freezing injury rhizomes frozen prior to excision, plants were cooled as described in the whole‐plant recovery test. After plants were thawed at 5°C for 24 h, a 3 cm section of rhizome associated with the newest fully developed tiller was harvested, cleaned to remove surface debris, rinsed three times in double distilled water and transferred to a test tube (1.3 × 10 cm) containing 1 ml of deionized water. To assess injury in rhizomes frozen following excision, tissue was excised from unfrozen plants and transferred to a capped test tube, cooled in a programmable freezer and thawed as described. Rhizomes frozen prior to or following excision were ice nucleated using frozen 100 μl droplets of MilliQ water.

An aliquot of 2 ml of MilliQ water was added to test tubes containing the intact and excised rhizomes. Tubes were transferred to a rotary shaker set at 100 rpm for 4 h. A 100 μl sample was quantified for initial conductivity (*I*_i_) with a LAQUAtwin‐EC‐33 conductivity meter (Horiba Instruments Inc., Kyoto, Japan). Samples were frozen in liquid nitrogen for 30 min, thawed at room temperature, placed on a rotary shaker (100 rpm for 8 h) and then re‐assessed to determine final conductivity (*I*_f_). Distilled water samples were included to assess the conductivity of the water sample (*I*_b_). A sub‐sample of tubes were exposed to liquid nitrogen prior to assessment of initial conductivity to assess the maximum level of injury in rhizomes. Percent injury (%Injury) as described by Sukumaran and Weiser (1972) was calculated as: [(*I*_i_ – *I*_b_)/(*I*_f_ – *I*_b_)]. 100. Relative electrolyte leakage as described by Lim et al. (1998) was calculated using the following formula: (%Injury_sample_/ %Injury_maximum_). 100.

### 2.3 Cold acclimation in AP13 and DAC6

To assess the physiological parameters at maximum freezing tolerance involving the most northern upland (DAC6) and southern lowland (AP13) genotypes, chamber temperature was cooled to 8/4°C with a photoperiod of 10 h. Switchgrass was sampled after 0, 7, 14, 21, 28, 35, 49 or 63 days to determine the temperature at which half of the switchgrass recovered from freezing injury (LT_50_) and rhizome water content as described below. The experiment was repeated four times for each genotype and treatment combination.

To assess rhizome water content, a 4 cm section of developing rhizome tissue collected from 10 plants was immediately weighed to obtain fresh mass (FM). Samples were dried at 60°C for 48 h and then re‐weighed to obtain the dry mass (DM). Rhizome water content was quantified as described by Willick et al. (2019) using the following formula: gH_2_O gDM^−1^ = (FM−DM)/DM.

### 2.4 Maximum freezing tolerance

A second experiment was conducted to assess the relationship between LT_50_ and the geographic origin of genotypes. Genotypes (Table 1) were placed in a growth cabinet for 21 days set at one of four different regimes: (1) 25/20°C and a 16 h photoperiod; (2) 25/20°C and a 10 h photoperiod; (3) 8/4°C and a 16 h photoperiod; (4) 8/4°C and a 10 h photoperiod. Switchgrass genotypes were then assessed for LT_50_ and the experiment was repeated four times for each genotype and treatment combination.

### 2.5 Threshold induction temperatures

Genotypes were exposed to a 16 or 10 h photoperiod and a constant temperature of 20°C, 16°C, 12°C, 8°C or 4°C for 7 days. Switchgrass exposed to each of the temperature and photoperiod treatments were grown under 70% relative humidity and a light intensity at the canopy level of 700 μmol photons m^−2^ s^−1^. All genotypes were assessed for survival after cooling to −8°C using the freezing tolerance protocol described above. The threshold induction temperature was recorded as the warmest acclimation temperature at which each genotype significantly enhanced survival above plants grown at a constant temperature of 20°C. Threshold induction temperature studies were repeated three times.

### 2.6 Climate data

We retrieved data for historical maximum and minimum mean surface temperatures (1971–2000) for the months of September, October, and November from WorldClim 2.0 data sets (Fick & Hijmans, 2017). All climate data was collected at the highest available resolution (30 arc s = 1 km^2^) and extracted at the site of origin for each genotype using QGIS version 3.24.0 (QGIS Core Development Team, 2021). Using QGIS, site or origin for each genotype was plotted over climate data displaying the historical mean air surface temperatures for September.

### 2.7 Statistical analyses

Statistical analyses were performed using R (version 4.2.1). Tests of significance to compare the differences in freezing injury from whole‐plant recovery, rhizomes harvested from frozen plants and rhizomes frozen independently were conducted by one‐way ANOVA (Type III) with the nlme package (Pinheiro et al., 2022). A Tukey's HSD test was performed with the agricolae package (de Mendiburu, 2021) to detect differences among the three treatments. In instances where there was no injury in the whole‐plant survival treatment, comparisons between intact and excised rhizomes were made with two‐tailed *t*‐tests. For the AP13 and DAC6 cold acclimation experiment, linear regression analysis was performed using the nlme and mgcv (Wood, 2022) packages to investigate the effects of the length of cold acclimation at 8/4°C on LT_50_ and rhizome water content. Linear models were initially fit based on each fixed explanatory variable separately, prior to fitting models at all fixed explanatory variables together. The Akaike information criterion was then used to select for the optimal model. A quadratic model was the most optimal model for the LT_50_ dataset (y = x + ax^2^) and a quartic model was the most optimal for water content dataset (y = x + ax^2^+ bx^3^). In each model the fixed independent variable (y) was days at 8/4°C and the dependent variable (x) was either LT_50_ or tissue water content.

Upland, coastal, and lowland maximum LT_50_ and threshold induction temperatures were modeled by generalized linear regression using the package lme4 (Bates et al., 2015), with genotype, latitude of origin and their interaction as fixed effects. Results from genotypes were pooled to assess the effect of photoperiod on threshold induction temperature or LT_50_ from ecotypes grown at 25/20°C or 8/4°C by ANOVA (Type I) with the car package (Fox et al., 2019). Tukey's HSD from the agricolae package was used to identify significant differences among treatments. Pearson's correlation coefficients were used to assess relationships between LT_50_, threshold induction temperatures, and historical mean air surface temperatures collected with QGIS from the genotype's latitude of origin.

## 3 RESULTS

### 3.1 Whole‐plant recovery provides the most consistent measure for assessing LT_50_

**FIGURE 2:**
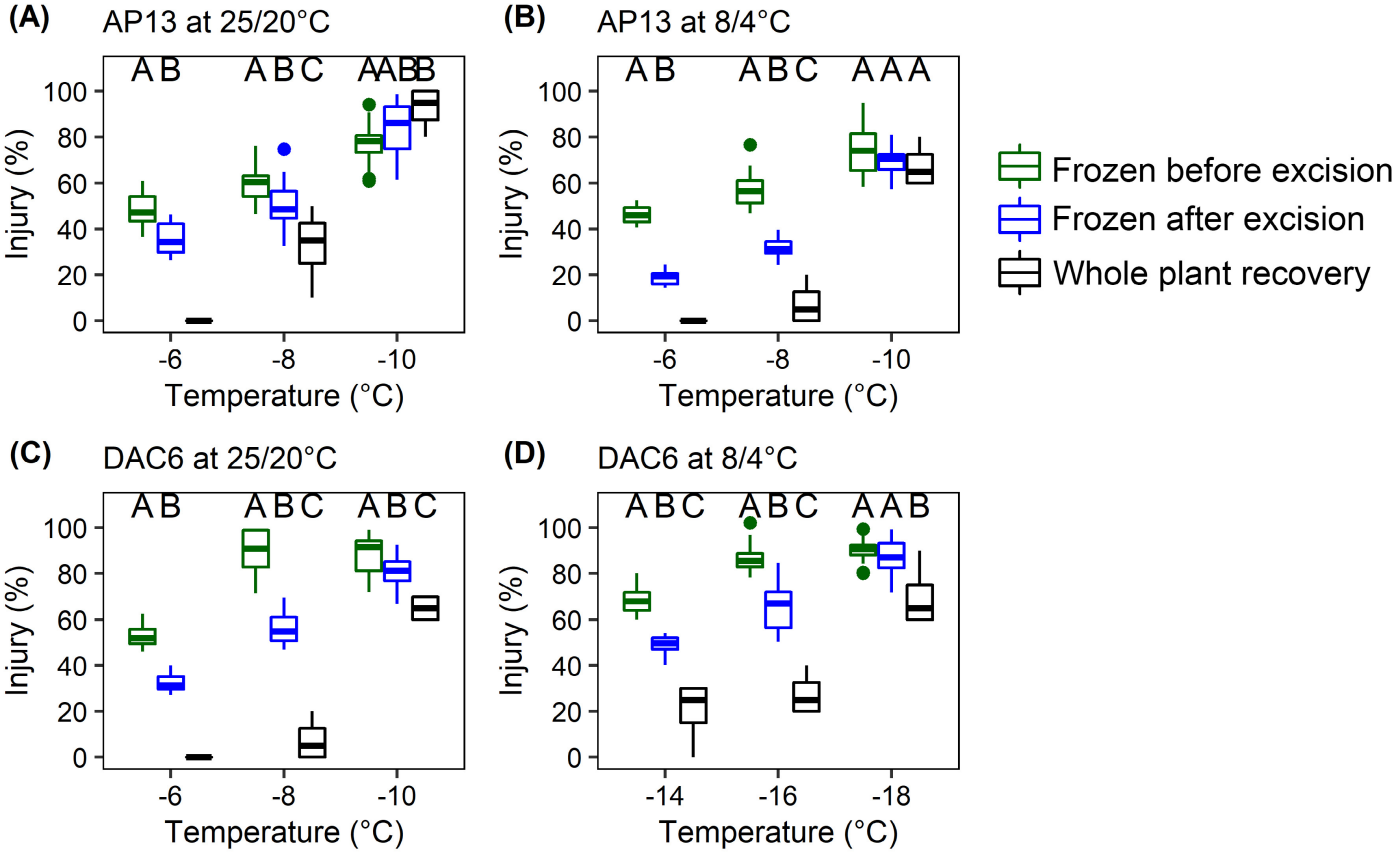
Comparison of injury calculated in switchgrass AP13 (A, B) and DAC6 (C, D) grown at 25/20°C or 8/4°C for 21 days. Injury was assessed by electrolyte leakage in rhizomes frozen prior to (green, *N* = 15) or following excision (blue, *N* = 15) and in whole plants freeze‐thawed and then assessed for recovery after 28 days (black, *N* = 5). Means within a sub‐zero temperature treatment followed by the same uppercase letter are not different based on Tukey's HSD (one‐way ANOVA [type III], *p* < 0.05). In instances where whole plants displayed no injury, significant differences within a sub‐zero temperature treatment between excised and intact rhizomes were based on a two‐tailed *t*‐test (*p* < 0.05).

Whole plants exposed to freeze–thaw conditions and then recovered over a 28 days period had a higher survival rate as compared with rhizomes frozen prior to or following excision (Figure 2). Rhizomes from AP13 cooled to −6°C or − 8°C following excision accrued more injury as compared with rhizomes cooled to the same temperatures prior to excision (−6°C, *t*‐test: *p* < 0.001; 8/4°C: *p* < 0.001) (−8°C, ANOVA 25/20°C: *p* < 0.001; 8/4°C: *p* < 0.001) (Figure 2A, B). The level of freezing injury did not differ among AP13 allowed to recover or rhizomes cooled to −10°C prior to or following excision (ANOVA, *p* = 0.173). The DAC6 genotype grown at 25/20°C or 8/4°C accrued less freezing injury if plants regrew for 28 days as opposed to rhziomes assessed for injury using the electrolyte leakage method (ANOVA, 25/20°C: *p* < 0.001, 8/4°C: *p* < 0.001) (Figure 2C, D). The whole‐plant LT_50_ of 25/20°C AP13 was −6.4°C and was reduced in assessed rhizomes frozen prior to or following excision by 1 and 2°C. Larger differences between freezing methods were observed in AP13 cold acclimated at 8/4°C for 21 days. Recovering AP13 developed an LT_50_ of −9.2°C which was reduced in rhizomes frozen prior to or following excision by 0.5 and 2.5°C. In DAC6 grown at 25/20°C, the LT_50_ of recovered plants was −9.3°C, which was reduced in rhizomes frozen prior to or following excision by 1.8 and 3.5°C. Cold acclimated DAC6 developed an LT_50_ of −16.7°C, which was 2.5 and 5.8°C cooler as compared with an LT_50_ assessed in rhizomes frozen prior to or following excision.

### 3.2 Exposure to 8/4°C lowers LT_50_ and rhizome water content in DAC6 to a greater amount than in AP13

**FIGURE 3:**
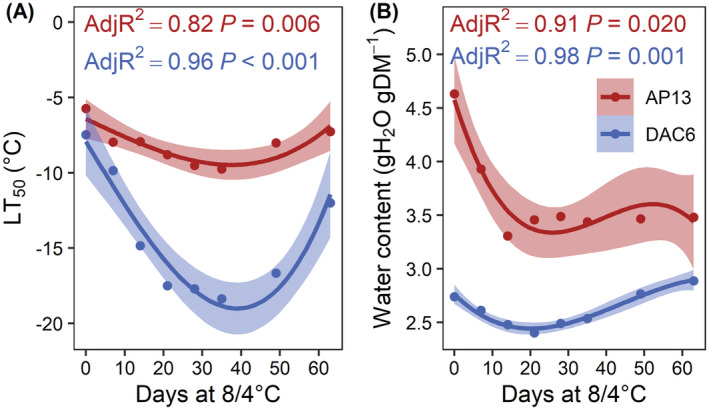
Switchgrass upland DAC6 (blue) and lowland AP13 (red) whole‐plant LT_50_ and rhizome water content when acclimated at 8/4°C with a 12 h photoperiod for 0 to 63 days. (A) The LT_50_ or lethal temperature at which 50% of the switchgrass pots recover. (B) Rhizome water content. Adjusted *R* ^2^ values indicate the fit of the regression lines. Shaded regions represent a 95% confidence interval on the fitted values. Data are presented as the means of four experiments in which each experiment consisted of samples collected from 10 pots.

For DAC6 and AP13, the LT_50_ and belowground tissue water content was positively correlated with the length of exposure to 8/4°C (DAC6: *p* < 0.001, AP13: *p* = 0.006) (Figure 3A). After 7 days at 8/4°C, there was a 2°C difference in the LT_50_ of DAC6 and AP13 (Figure 2A). Acquisition of freezing tolerance as measured by LT_50_ markedly increased during the first 21 days at 8/4°C (Figure 3A). Between 28 and 35 days, the rate of cold acclimation declined to produce a curvilinear relationship between the LT_50_ and the number of days grown at 8/4°C. The maximum LT_50_ was attained after 21 days for DAC6 (LT_50_ = −18°C) and after 28 days for AP13 (LT_50_ = −9°C). For both genotypes, a loss in LT_50_ was first observed after 49 days at 8/4°C.

Prior to cold acclimation, AP13 belowground tissues contained 1.1 gH_2_O gDM^−1^ more tissue water in comparison with DAC6 (Figure 3B). The accruement of freezing tolerance over the first 21 days after exposure to 8/4°C corresponded with a decline in rhizome water content (DAC6: *p* = 0.001, AP13: *p* = 0.020). Belowground tissue collected after 21 days at 8/4°C from DAC6 contained 0.4 g H_2_O gDM^−1^ and from AP13 1.1 gH_2_O gDM^−1^ less as compared with the similar tissues harvested from switchgrass grown at 25/20°C. This loss in water content after 21 days at 8/4°C does not reflect that the belowground tissue of AP13 still maintained 1.0 gH_2_O gDM^−1^ more tissue water in comparison with DAC6. The gradual loss in LT_50_ after 49 days exposure to 8/4°C (Figure 3A) corresponded with the accumulation of rhizome tissue water (Figure 3B). Rhizome dry matter accumulation did not vary among AP13 (ANOVA, *F* = 0.293, *p* = 0.589, *N* = 15) or DAC6 (ANOVA, *F* = 3.848, *p* = 0.052, *N* = 15) exposed to 8/4°C and, overall, DAC6 = 32.6 ± 2.0 mg (*N* = 120; all plants pooled) and AP13 = 49.9 ± 3.4 mg (*N* = 120; all plants pooled).

### 3.3 Temperature influences genotype LT_50_ and the induction of acclimation more than photoperiod

Reducing the photoperiod by 6 h in switchgrass grown 25/20°C significantly enhanced the mean LT_50_ across lowland and coastal switchgrass genotypes by 1.2°C and upland switchgrass genotypes by 1.0°C (Table 2, *p* < 0.001). Shifting the photoperiod from 16 to 10 h for switchgrass grown at 8/4°C was ineffective in enhancing the LT_50_ of upland, coastal and lowland genotypes (*p* = 0.342). Upland genotypes grown at 8/4°C developed a 3.7°C to 4.0°C lower LT_50_ as compared with the coastal genotypes and a 4.9°C to 5.0°C lower LT_50_ as compared with the lowland genotypes. Interestingly, shifting the photoperiod from 16 to 10 h raised the cold acclimation threshold induction temperature by 1.3°C among the upland genotypes (*p* < 0.001). A similar shift in threshold induction temperature was not observed in the coastal and lowland genotypes (*p* > 0.05). Cold acclimation was induced in upland genotypes at a 4.0°C to 4.1°C higher temperature as compared with the coastal genotypes and a 6.1°C to 7.4°C higher temperature as compared with the lowland genotypes.

**TABLE 2:** Mean values for LT_50_ after 21 days and the cold acclimation threshold induction temperatures for eight upland, three coastal, and five lowland switchgrass genotypes.

Ecotype	Photoperiod (h)	LT_50_ at 25/20°C (°C)	LT_50_ at 8/4°C (°C)	Induction (°C)
Upland	16	−7.4 ± 0.1A	−13.1 ± 0.8B	13.9 ± 0.9B
	10	−8.4 ± 0.1B	−14.0 ± 0.8B	15.2 ± 0.9A
Coastal	16	−7.2 ± 0.1A	−9.4 ± 1.2A	9.9 ± 2.2C
	10	−8.4 ± 0.1B	−10.0 ± 1.6A	10.3 ± 3.0C
Lowland	16	−7.3 ± 0.1A	−8.2 ± 0.8A	7.8 ± 0.7D
	10	−8.5 ± 0.1B	−9.0 ± 0.9A	7.8 ± 0.9D
	Ecotype (E)	0.711	<0.001	<0.001
	Photoperiod (P)	<0.001	0.342	0.011
	E × P	0.363	0.993	0.134

*Note*: Means within a column followed by the same uppercase letter are not different based on Tukey's HSD (two‐way ANOVA [type III], *p* < 0.05). The LT_50_ (upland, *N* = 32; coastal, *N* = 12; lowland, *N* = 20) and cold acclimation induction temperature (upland, *N* = 24; coastal, *N* = 9; lowland, *N* = 15) data are presented as the means of eight upland, three coastal or five lowland genotypes.

### 3.4 Northern adapted genotypes develop a lower induction temperature for cold as compared with more southern genotypes

**FIGURE 4:**
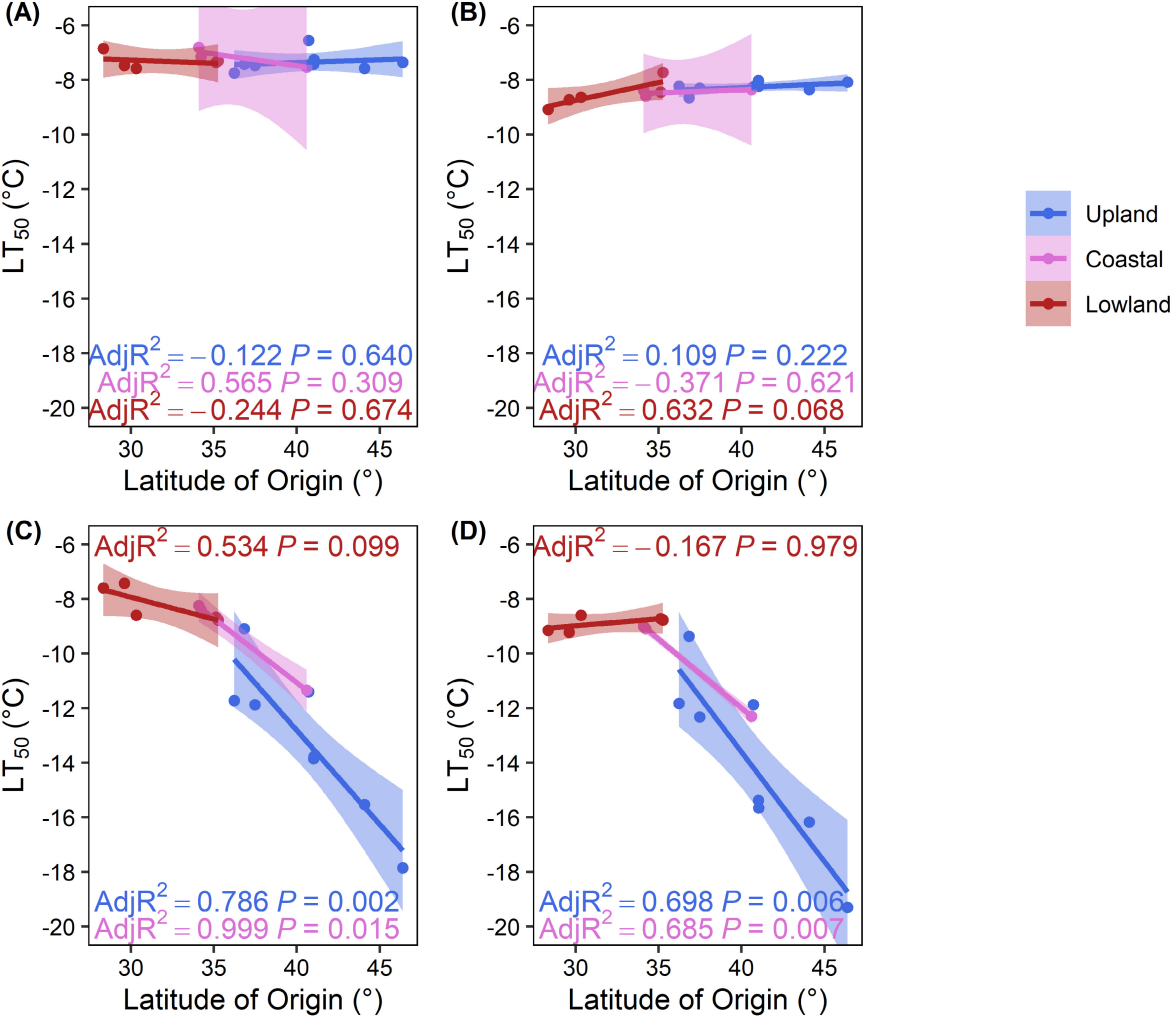
Lethal temperature at which half of the plants were able to recover from freezing injury (LT_50_) in upland (blue), coastal (purple), and lowland (red) switchgrass genotypes based on their latitude of origin. Linear regression analyses of the cold acclimation induction temperature and latitude of origin in switchgrass when grown for 21 days at (A) 25/20°C with a 16 h photoperiod; (B) 25/20°C with a 10 h photoperiod; (C) 8/4°C with a 16 h photoperiod; (D) 8/4°C with a 10 h photoperiod. Adjusted *R* ^2^ values indicate the fit of the linear regression lines. Shaded regions represent a 95% confidence interval on the fitted values. Each point represents the mean of four independent experiments.

Among the upland and coastal genotypes, significant differences in LT_50_ were observed due to latitude of origin (*p* < 0.001), acclimation temperature (*p* < 0.001), photoperiod (*p* < 0.001), and an interactive effect between latitude and temperature (*p* < 0.001) (Table 3). There were significant differences in LT_50_ were observed among lowland genotypes due to acclimation temperature (*p* < 0.001), photoperiod (*p* < 0.001) and interactions among latitude and temperature (*p* < 0.01), latitude and photoperiod (*p* < 0.05), as well as temperature and photoperiod (*p* < 0.001). When switchgrass genotypes were grown at 25/20°C with a 10 or 16 h photoperiod there was no relationship between the LT_50_ and latitude of origin (Figure 4A, B). Upland and coastal genotypes grown at 8/4°C with a 10 and 16 h photoperiod developed a linear relationship between the LT_50_ and latitude of origin (16 h upland: *p* = 0.02; 16 h coastal: *p* = 0.015; 10 h upland: *p* = 0.006; 10 h coastal: *p* = 0.007) (Figure 4C, D).

**TABLE 3:** Analysis of variance of latitude of origin, temperature (25/20°C, 8/4°C), and photoperiod (16 h, 10 h) effects on the LT_50_ in upland, coastal, and lowland switchgrass

	Upland		Coastal		Lowland	
Effect	df	*F*	df	*F*	df	*F*
Latitude (L)	1, 120	190.4***	1, 40	66.0***	1, 72	0.1
Temperature (T)	1, 120	1103.1***	1, 40	99.0***	1, 72	31.6***
Photoperiod (P)	1, 120	24.3***	1, 40	28.7***	1, 72	69.0***
L × T	1, 120	227.2***	1, 40	50.5***	1, 72	7.8**
T × P	1, 120	1.5	1, 40	0.3	1, 72	21.7***
L × P	1, 120	0.0	1, 40	1.5	1, 72	5.4*
L × T × P	1, 120	0.9	1, 40	1.2	1, 72	0.6

*Note*: *F*‐values with ‘*’, ‘**’, and ‘***’ are significant at *p* < 0.05, *p* < 0.01 and *p* < 0.001, respectively.

**FIGURE 5:**
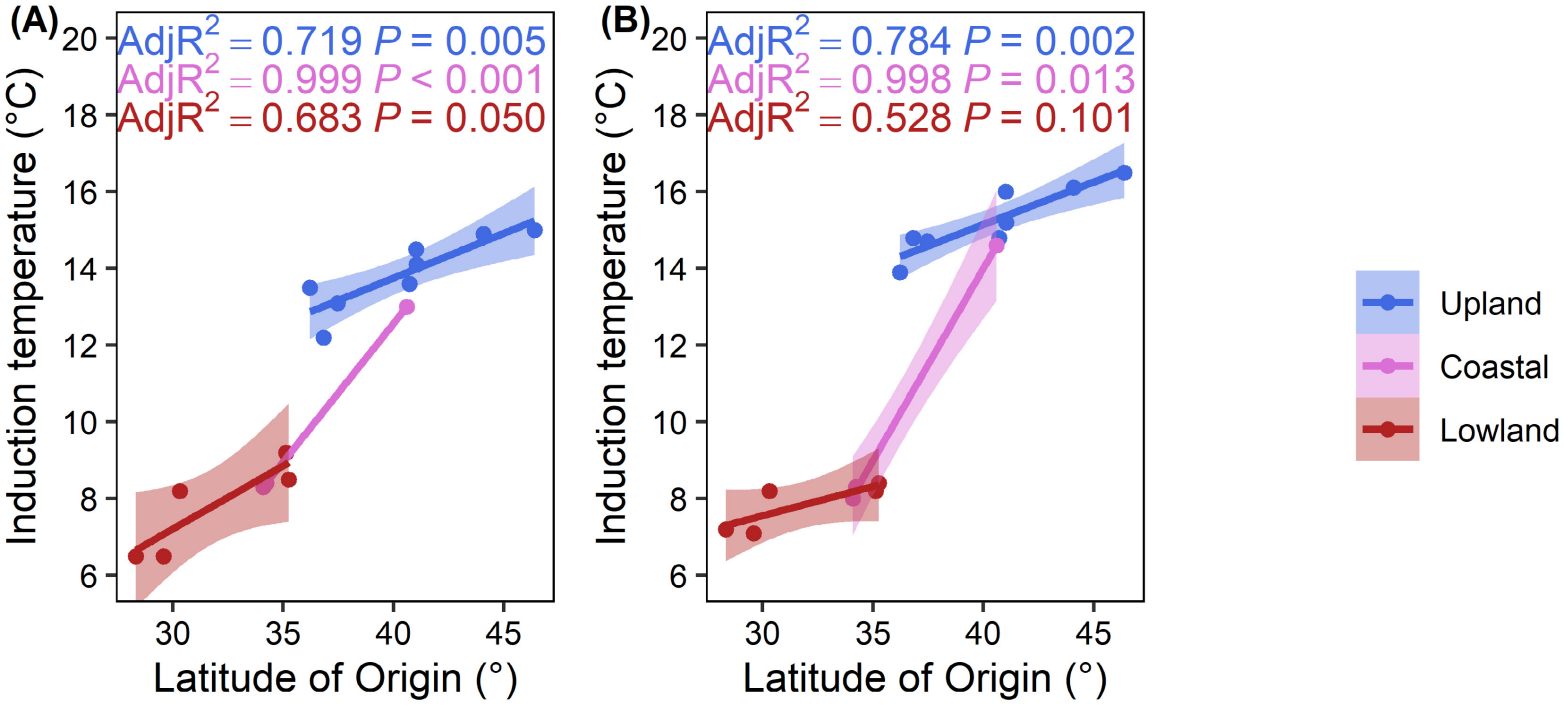
Threshold temperatures required to induce a cold acclimation response in upland (blue), coastal (purple), and lowland (red) switchgrass genotypes. Linear regression analyses of the cold acclimation induction temperature and latitude of origin in switchgrass when grown under a (A) 16 h or (B) 10 h photoperiod. Adjusted *R* ^2^ values indicate the fit of the linear regression lines. Shaded regions represent a 95% confidence interval on the fitted values. Each point represents the mean of three independent experiments.

For upland and coastal genotypes, there was a significant effect of latitude (*p* < 0.001) and for upland genotypes an interactive effect of latitude and photoperiod (*p* < 0.001) on threshold induction temperatures (Table 4). Interestingly in lowland genotypes, the latitude of origin (*p* = 0.424) and photoperiod (*p* = 0.894) did not influence the threshold induction temperatures (Table 4). There was a significant linear relationship when upland and coastal genotypes were grown under a 10 and 16 h photoperiod and lowland genotypes grown under a 16 h photoperiod between the cold acclimation threshold induction temperature and the latitude of origin (16 h upland: *p* = 0.005; 16 h coastal: *p* < 0.001; 16 h lowland: *p* = 0.050; 10 h upland: *p* = 0.002; 10 h coastal: *p* = 0.013) (Figure 5).

**TABLE 4:** Analysis of variance summary table evaluating the effects of photoperiod (16 h, 10 h) and latitude of origin on the threshold induction temperatures of upland, coastal, and lowland switchgrass.

	Upland		Coastal		Lowland	
Effect	df	*F*	df	*F*	df	*F*
Latitude (L)	1, 44	54.92***	1, 14	99.57***	1, 26	0.66
Photoperiod (P)	1, 44	3.29	1, 14	0.70	1, 26	0.02
L x P	1, 44	7.52**	1, 14	2.93	1, 26	0.03

*Note*: *F*‐values with ‘*’, ‘**’, and ‘***’ are significant at *p* < 0.05, *p* < 0.01 and *p* < 0.001, respectively.

### 3.5 Climatic influence at the latitude of origin on LT_50_ and induction temperature

Regardless of photoperiod, upland and coastal genotypes exposed to 8/4°C developed an LT_50_ that was correlated with higher latitudes (*p* < 0.01) as well as minimum and maximum air temperatures (Table 5). Threshold induction temperatures in upland and coastal genotypes grown at 10 or 16 h photoperiods were correlated with latitude (*p* < 0.01), minimum air temperature at the latitude of origin in September (*p* < 0.01), minimum and maximum air temperatures at the latitude of origin in November (*p* < 0.05). Upland genotypes also developed threshold induction temperatures when grown at 10 or 16 h photoperiods that correlated with the maximum air temperatures at the latitude of origin in September (*p* < 0.01). Lowland genotypes did not develop a correlation between LT_50_ and latitude (*p* > 0.05) Interestingly, there was a correlation between the threshold induction temperatures reported in lowland genotypes grown under a 16 h photoperiod with latitude and all minimum and maximum temperatures at the latitude of origin except for minimum temperatures in October (*p* < 0.05) (Table 5).

**TABLE 5:** Correlations between upland (*N* = 8), coastal (*N* = 3), and lowland (*N* = 5) switchgrass freezing tolerance and cold acclimation threshold induction temperatures in comparison with site of origin fall temperature climate conditions

	LAT (°)	LON (°)	Max_Sep_ (°C)	Min_Sep_ (°C)	Max_Oct_ (°C)	Min_Oct_ (°C)	Max_Nov_ (°C)	Min_Nov_ (°C)
Upland								
LT_50_ LDW (°C)	0.197	−0.113	−0.240	−0.299	−0.341	−0.036	−0.068	−0.262
LT_50_ SDW (°C)	0.487	0.339	−0.501	−0.411	−0.260	−0.572	−0.549	−0.417
LT_50_ LDC (°C)	−0.904	−0.117	0.858	0.750	0.583	0.782	0.878	0.836
LT_50_ SDC (°C)	−0.900	−0.230	0.814	0.692	0.519	0.825	0.896	0.801
TID LD (°C)	0.872	0.278	−0.794	−0.649	−0.463	−0.899	−0.941	−0.789
TID SD (°C)	0.903	0.189	−0.748	−0.641	−0.508	−0.785	−0.873	−0.806
Coastal								
LT_50_ LDW (°C)	−0.884	−0.999	0.693	0.620	0.579	0.896	0.845	0.813
LT_50_ LDC (°C)	0.562	0.142	−0.793	−0.848	−0.874	−0.541	−0.625	−0.669
LT_50_ SDW (°C)	−1.000	−0.910	0.941	0.904	0.881	1.000	0.995	0.987
LT_50_ SDC (°C)	−1.000	−0.903	0.947	0.911	0.889	1.000	0.996	0.989
TID LD (°C)	1.000	0.899	−0.949	−0.914	−0.892	−1.000	−0.997	−0.991
TID SD (°C)	1.000	0.908	−0.942	−0.905	−0.882	−1.000	−0.995	−0.987
Lowland								
LT_50_ LDW (°C)	−0.259	−0.017	0.347	0.334	0.367	0.575	0.503	0.467
LT_50_ LDC (°C)	0.851	0.794	−0.862	−0.86	−0.856	−0.852	−0.858	−0.858
LT_50_ SDW (°C)	−0.807	−0.811	0.843	0.828	0.823	0.798	0.807	0.822
LT_50_ SDC (°C)	0.603	0.594	−0.661	−0.640	−0.641	−0.656	−0.648	−0.659
TID LD (°C)	0.873	0.848	−0.900	−0.890	−0.891	−0.862	−0.875	−0.888
TID SD (°C)	0.804	0.799	−0.843	−0.827	−0.824	−0.809	−0.814	−0.826

*Note*: Significant correlations are in light gray (*p* < 0.05) and dark gray (*p* < 0.01).

Abbreviations: LDC, long day (16 h) photoperiod at 8/4°C; LDW, long day (16 h) photoperiod at 25/20°C; LT_50_, temperature at which half the plants recovered from freezing injury; SDC, short day (10 h) photoperiod at 8/4°C; SDW, short day (10 h) photoperiod at 25/20°C; TID, threshold induction temperature.

## 4 DISCUSSION

In this study, we found evidence supporting our hypothesis that upland switchgrass develops a higher degree of freezing tolerance through cold acclimation than coastal and lowland switchgrass genotypes. Following identification of the optimal method to assess freezing tolerance and length of cold acclimation in upland DAC6 and lowland AP13, we then found that more northern genotypes within each ecotype initiated cold acclimation at higher threshold temperatures. Furthermore, in upland and coastal genotypes we observed a strong north to south latitudinal relationship in maximum freezing tolerance following cold acclimation. However, the magnitude of this latitudinal relationship was strikingly different for upland and coastal switchgrass (Figure 4). Beyond temperature, photoperiod had a moderate effect on cold acclimation. For upland genotypes, a 10 h photoperiod resulted in a 1.3°C higher threshold temperature required to initiate cold acclimation as compared with plants grown under a 16 h photoperiod. When upland, coastal, and lowland genotypes were grown under 25/20°C, exposure to a 10 h photoperiod did enhance the maximum freezing tolerance. Cooler fall air temperatures at the latitude of origin correlated with maximum freezing tolerance and threshold induction temperatures. Overall, these results suggest that upland genotypes, especially far northern ones, have evolved a response to cooling temperatures earlier in the fall than coastal and lowland genotypes. The additional time for cold acclimation by northern upland genotypes coupled with their greater freezing tolerance following acclimation, provides them with a competitive advantage in overwinter survival.

### 4.1 Method of freezing and length of cold acclimation influences switchgrass LT_50_

Recovered switchgrass developed a lower LT_50_ as compared with rhizomes from the same genotype that were frozen prior to or following excision, which suggests that the electrolyte leakage assay of excised tissues may not accurately reflect freezing injury observed in whole switchgrass plants. Chen et al. (1983) reported that root initiation in the winter wheat overwintering crown organ was impaired after freezing injury. Tanino and McKersie (1985) visually demonstrated using a tetrazolium chloride vitality assay that if injury was severe enough in the vascular tissues at the base of the crown, then it would impede whole‐plant recovery. Cell death was observable in freeze‐thawed crown tissues up to 3 days after exposure to regrowth temperatures (Willick et al., 2018). These studies suggest that injury in belowground overwintering organs from freezing injury first manifests as an inability to develop new roots and then as cell death in the region of the vascular transition zone and later the apical meristem. An electrolyte leakage based‐assay in freeze‐thawed rhizomes would only detect injury after rupture of the cell membranes. Rhizomes may also have a different regrowth pattern than crowns that may reduce the usefulness of the electrolyte leakage assay as an alternative for assessing recovery. Schwartz and Reaney (1989) observed that desert saltgrass (*Distichlis stricta* Torr.) and alkali cordgrass (*Spartina gracilis* Trin.) sustained significant injury to sections of rhizome and regrew from small tissues likely associated with meristem regions. Since large vascularized tissues surrounding meristem regions contribute a higher concentration of leaked electrolytes in comparison with the uninjured smaller meristem tissues (Willick et al., 2019), then it is likely that the electrolyte leakage assay is not appropriate for measuring injury in switchgrass rhizomes.

In our study, the difference between electrolyte leakage LT_50_ observed in rhizomes frozen prior to or following excision (Figure 2) may suggest the bud meristems within the rhizome are protected by a yet unknown organ‐level ice segregation mechanism (Sakai & Larcher, 1987). In addition, the excision site of the rhizome may serve as a site for intrinsic ice nucleation and promote extracellular freezing. Although the electrolyte leakage‐based LT_50_ may express the relative resistance of rhizome tissue to freezing, the technique may not focus on the critical tissues within the overwintering organ essential for plant survival. Freezing rhizomes after their excision from the plant may also introduce an artifact into the freezing process (Peixoto & Sage, 2016). We elected for these reasons to use the whole‐plant recovery assay for the subsequent experiments.

### 4.2 Switchgrass overwintering through the induction of cold acclimation versus enhancing the maximum freezing tolerance

In temperate regions, overwintering plants undergo periodic transitions from lower to higher levels of tolerance to freezing injury. This generally results in two different mechanisms of acclimation. In the first instance, plants that have adapted to climates with a mild winter or those that do not experience severe frost events acquire greater resistance as a direct consequence of the decline in temperature (Sakai & Larcher, 1987). Alternatively, there is a level of periodicity observed in plants grown in alpine and temperate regions that develop aboveground woody tissues or belowground overwintering storage organs (Sakai & Larcher, 1987). In these instances, a gradual transition to developmental arrest commences in the fall and by spring the plants begin to develop new growth (Chang et al., 2021; Sarath et al., 2014).

In switchgrass, we observed that this seasonal periodicity is triggered by threshold temperatures that can be modulated by a short‐day photoperiod. Cold acclimation can initiate at higher temperatures under a short (10 h) as compared with switchgrass grown under a long‐day (16 h) photoperiod. Furthermore, we did not observe a difference in the length of cold acclimation required to achieve maximum freezing tolerance between AP13 and DAC6. Cold acclimation at 8/4°C and a 10 h photoperiod between 21 and 49 days (Figure 3) was comparable to lengths of time required to attain maximum freezing tolerance in North American winter cereals (Fowler et al., 1981; Willick et al., 2019) and field‐grown populations of upland switchgrass (Hope & McElroy, 1990).

Our observations (Figure 3B) further support previous reports that the lower LT_50_ of the critical belowground perennating organs corresponded with the loss of tissue water (Clifton‐Brown & Lewandowski, 2000; Willick et al., 2019). Differences in DAC6 and AP13 LT_50_ could be explained by total rhizome water content. Generally, within a species, critical overwintering organs that accumulate lower volumes of tissue water are more capable of surviving more severe forms of freezing injury (Fowler et al., 1981). The loss of freezing tolerance and accumulation of rhizome water at 49 days of exposure to 8/4°C corresponds with previous observations in winter wheat and rye crowns (Fowler, 2008; Willick et al., 2019) and could result from the loss of cryoprotective sugars reconstituted by the switchgrass to repair chill‐induced injury. Further experimentation is needed to determine whether rhizome water content would be a useful screening method for genotypic‐level freezing tolerance.

Results presented in our study also imply that the overall cold acclimation pattern is similar between patterns previously reported in trees (Howe et al., 1995; Li et al., 2003; Tanino et al., 2010) and the perennial grass *Arrhenatherum elatus* (Malyshev et al., 2014). Genotypes within the upland ecotype could acclimate to a greater degree than the coastal, and lowland genotypes (Table 3, Figure 4), indicating that the upland genotypes are better adapted to overwinter at higher latitudes. In contrast with our observations in lowland and coastal switchgrass genotypes that only obtained maximum freezing tolerance in response to reduced temperature, the Southern ecotypes of *A. elatus* (Malyshev et al., 2014) and black cottonwood (*Populus trichocarpa* Torr. & Gray) (Howe et al., 1995) require shorter photoperiods to achieve similar cold acclimation levels as the northern ecotypes. In agreement with our observations in upland switchgrass, the northern ecotypes of *A. elatus* and woody perennials are generally more responsive to declining photoperiod and temperature (Howe et al., 1995; Li et al., 2003; Malyshev et al., 2014; Tanino et al., 2010).

Cold acclimation in herbaceous plants was historically believed to initiate between 5°C (Sakai & Larcher, 1987) and 10°C (Olien, 1967). Our observations suggest that upland switchgrass genotypes initiate cold acclimation at temperatures between 16°C and 10°C (Table 4, Figure 5), which is consistent with observations from freezing tolerant winter cereals (Fowler et al., 1999; Fowler, 2008; Wilen et al., 1997). A higher induction temperature for cold acclimation in winter wheat and fall rye corresponded with greater maximum freezing tolerance (Fowler et al., 1999; Fowler, 2008; Wilen et al., 1997). In our study, the most northern upland switchgrass DAC6 cold acclimates after exposure to 15°C (Figure 5), which is comparable to the highest cold acclimation temperature observed in winter wheat cv. Norstar (Fowler, 2008). Unlike red osier dogwood (*Cornus sericea* L.) that acclimates at 20/15°C under a 10 h but not a 16 h photoperiod (Fuchigami et al., 1971), the switchgrass genotypes assessed in our study required exposure to low threshold temperatures to induce cold acclimation (Figure 5). While upland genotypes grown under a 10 h as opposed to 16 h photoperiod can induce cold acclimation at higher temperatures, exposure to prolonged low temperatures (8/4°C) is still required to attain maximum levels of freezing tolerance.

### 4.3 Cooler fall temperatures at the latitude of origin correspond with enhanced freezing tolerance and higher threshold induction temperatures

Out of the climatic parameters used to characterize the latitudes of origin, minimum surface temperatures in October, and surface temperatures in November were good predictors for threshold induction temperatures in coastal and upland switchgrass (Table 5). These observations further support our observations that the more northern genotypes of switchgrass can initiate cold acclimation at higher temperatures and obtain a lower LT_50_ as compared with more southern genotypes. Our observations support earlier trends reported in a common garden experiment using red osier dogwood northern ecotypes ranging from the North Dakota to Alaska, USA that cold acclimated earlier in the fall as compared with a coastal ecotype that enhanced its freezing tolerance only after exposure to the first fall frost (Smithberg & Weiser, 1968). This indicates that, while certain ecotypes and genotypes within an ecotype are better equipped to grow under extended summers at coastal or southern latitudes, other ecotypes have adapted to shorter growing seasons and require the ability to perceive earlier seasonal shifts in temperature.

## 5 CONCLUSIONS

We have demonstrated differences in LT_50_ and cold acclimation threshold induction temperatures for a perennial grass species. Compared with the coastal and lowland ecotype, the upland ecotype was more responsive to short days and low temperatures, resulting in the earlier initiation of cold acclimation and the development of a higher maximum level of freezing tolerance. Exposure to low threshold temperatures was essential to attain maximum freezing tolerance. We also observed a relationship between the latitude of origin among the tested upland and coastal genotypes, the LT_50_ and the cold acclimation threshold induction temperature. Northern genotypes within an ecotype induce a cold acclimation response at a higher threshold temperature and attain a greater maximum LT_50_ as compared with more southern genotypes within an ecotype. From an applied standpoint, the results of this study indicate that this wide range in threshold induction temperatures likely provides the more freezing‐tolerant genotypes with a much longer time to prepare for winter. Higher induction temperatures could be an adaptive mechanism used to enhance tolerance against early fall frosts more prevalent at northern latitudes. This will further promote survival in future climate scenarios where extreme shifts in temperature become more prevalent at the beginning and end of the growing season.

## AUTHOR CONTRIBUTIONS

Ian Willick conceived the study, performed the experiments, analyzed, and interpreted the data. David Lowry supervised the experiments and assisted in data analysis and interpretation. Ian Willick wrote the manuscript with input from David Lowry. Both authors contributed to editing and the final preparation of the manuscript.

## Data Availability

Data are accessible through Dryad. (doi: 10.5061/dryad.5tb2rbp6p; Willick and Lowry, 2022).
